# Introduction: ‘Why is there persistent disease despite aggressive therapy of rheumatoid arthritis?’

**DOI:** 10.1186/ar4592

**Published:** 2014-06-26

**Authors:** Pierre Miossec

**Affiliations:** 1 Immunogenomics and Inflammation Research Unit EA 4130, University of Lyon 1, Department of Immunology and Rheumatology, Hôpital Edouard Herriot, 69437 Lyon, Cedex 03, France

## 

During the last 20 years, obvious progress has been made in the care of patients with rheumatoid arthritis (RA). This results from the combination of better diagnosis, earlier use of classic drugs, and the introduction of new targeted therapies. Despite these positive results, RA remains a chronic disease and induction of remission is still rarely obtained [1]. It is as if RA were a series of lost battles. More recently, although RA care improved, chronic inflammation was shown to have an effect on mortality, specifically from cardiovascular events.

The goal of this series of short articles on RA chronicity is to give readers of *Arthritis Research & Therapy* the views of leading experts in the field. This combines lessons coming from daily clinical observations to a better understanding of basic molecular changes in mesenchymal cells.

## A question of definition

RA is a heterogeneous disease, as reflected by the various degrees of joint destruction and disability between patients. The complexity of such heterogeneity has been increasing over time with more markers, from autoantibodies and human leukocyte antigen (HLA) subtypes to extensive genome-wide polymorphisms and serological markers. Use of these markers has been helpful at a statistical level to compare populations but much less so to predict severity for a given patient.

If it is not possible to have a simple definition of a disease, we could mix different entities under the same definition. This is strongly stated by Gary Firestein, who proposes that we now need to consider RA a syndrome in order to include the different etiologies, clinical presentation and severity, and response rates to treatment [2].

If RA is now a syndrome, how do we use diagnostic criteria? Here again these criteria are indeed useful to compare populations but, even updated, they have limitations at the level of an individual patient. As a consequence of their (too) strict use, the associated risk is then the delay needed to fulfill enough of those criteria so that the diagnosis can be made.

## Early and late rheumatoid arthritis: two different stories

Clinical experience clearly shows that acting early is the best way to control disease and induce remission. Furthermore, the combination of disease-modifying anti-rheumatic drugs is more effective including anti-TNF and methotrexate (MTX) at all stages of RA in patients in whom low disease activity is not achieved on monotherapy with MTX. The ‘treat to target’ strategy for optimal control of disease has been an important advance in RA care.

Emery [3] rightly advocates that with RA we are always far too late and too often miss induction of remission. Although it makes sense to act early, we now better understand the underlying mechanisms. At an early stage, control of inflammation has been shown to be very effective. This is the stage at which the disease is driven by the classic RA lesion with migration of inflammatory cells from blood leading to ectopic lymphoid accumulation in the synovium, looking like an activated lymph node. Of interest also is the fact that the bone marrow of juxta-articular bone shows the same pattern, at the site of bone edema [4]. Thus the question is: is the process coming from the synovium then attacking bone, or is it the other way around? Emery proposes the use of the most effective drugs alone or even more in combination at this stage to prevent or reduce the risk of moving to the stage of chronic RA.

This group of patients with chronic RA consists of those included in clinical trials to test new treatments. Most of these patients have failed to respond to at least one TNF inhibitor. Disease has been present for many years and destruction is obvious. This situation is typical of another lost battle. Once again it can be predicted that many of these patients will not respond to immune-mediated therapies, because the disease is not driven by TNF, interleukin-6, T cells, or B cells anymore (or has never been driven) by these pathways. Even in the responders, chronicity is unfortunately still there, as easily shown by relapse when stopping or even just reducing treatment [5]. Treatment is as chronic as the disease. Worse, over time, different pathways are now activated because of prolonged inhibition of treatment-targeted ones. Thus, blocking the TNF pathway can lead to activation of the T helper 17 (Th17) pathway in patients losing response [6]. Lack of response could result first from the low expression of the target. It makes sense that patients responding to TNF should have a TNF-driven disease. Detection of functional TNF in those patients is in line with this hypothesis [7]. Lack of enough specificity could also contribute to a mixed response. Nistala and Mauri [8] indicate that the use of an anti-CD20 antibody by depleting all CD20-positive B cells can also eliminate the beneficial regulatory B cells.

## Mesenchymal cells: the forgotten target

Not only does chronic synovium infiltrate lead to the inflammatory clinical picture but its chronicity induces changes in resident cells, namely mesenchymal cells such as synoviocytes in the synovium but also in lymph nodes and bone marrow. When such damage has occurred, increased survival of these modified cells will now induce a self-perpetuating disease in the absence of the initial inflammation.

Epigenetic studies on mesenchymal cells, specifically synoviocytes, have clearly indicated that cells from the RA joint are different from cells from normal and osteoarthritis synovium [9]. Frank-Bertoncelj and Gay [10] advocate, and have been advocating for years, that acting at this late stage on inflammation and immune targets is useless. Instead, the modified synoviocytes should be the target. Years ago, this was done with radioactive or surgical synovectomy in a non-specific manner. Today new strategies need to be developed to target those cells through metabolic intervention, as in cancer, or their physical elimination or both. One limitation is to find ways to target specifically those cells known to lack markers.

To further support the concept of early intervention, it is possible that such changes in mesenchymal cells occur rather quickly. At least *in vitro*, exposure of synoviocytes to a combination of proinflammatory cytokines for a few days is enough to induce such changes, making these cells less sensitive to apoptotic signals.

## The pre-clinical phase, prevention, and eradication

Studies using samples collected years before the clinical signs appear have indicated that normal individuals who will, or at least could, develop RA show signs of immune activity years before. Appearance of autoantibodies and increased levels of proinflammatory cytokines have been described [11]. These abnormalities become more common when the disease is ready to start. However, the rate of RA is too low to justify detection of these markers even in a selected, otherwise healthy population.

Exogenous and endogenous inducers have been identified. Oral infection leads to parodontopathies. *Porphyromonas gingivalis* has been identified as an important target because it can induce changes in proteins, making them (auto)antigenic. However, again reflecting the heterogeneity, other mechanisms have been proposed, including lung inflammation possibly through smoking and changes in the bacterial microbiote. These later changes in the intestinal microbiote are starting to be identified, possibly leading to disease initiation through changes in the T-cell phenotype toward a more inflammatory Th17 pathway [12].

Because of such a long list, it is difficult today to select a simple way to prevent RA. However, this needs to be kept in mind, as it will be the best way to eliminate the disease. Large studies could demonstrate the influence of better hygiene and more specifically if vaccination against a pathogen could have an effect on RA incidence years later. If the origin of RA is caused simply by a single infectious agent, then the disease should disappear. Given the proven association of cigarette smoking in the causation of RA via induction of anti-cyclic citrullinated peptide in HLA-susceptible individuals, cessation of smoking remains the only plausible preventative strategy [13].

At the undifferentiated arthritis stage, the patients complaining of arthralgias may have transient swelling, but, when these patients are seen, joint examination is usually normal. Current markers are not very useful. This is where the first battle can be lost. There are two options: do we wait and see, or do we act quickly? This would imply treatment with drugs with a good safety efficacy balance. In that case, who should prescribe: the primary care physician or the specialist? If the latter, we would need more specialists with rapid access. At the end, this is, and remains, a health-system decision. As daily clinical practice indicates, acting at that stage is the best way to prevent extension and to allow drug-free remission. Maybe the risk is to treat some patients who are not going to develop RA. However, the net benefit is to avoid seeing patients at too late a stage.

## Proposal for action

If RA is a series of lost battles, getting ready and having the right protection are the way to go. Above all, the general population needs to be aware of the risk of inflammatory joint complaints. Having access early to qualified care and treatment is, and will remain, the best way to improve the outcome.

Modalities of the clinical trials have to be modified, possibly transferring those used in cancer care, with early combinations of drugs, followed by simplification when remission is obtained to drug-free follow-up. Targeting needs to be adjusted to the stage of the disease, possibly using a combination regimen at the start. This should include new targets beyond the classic immune-related ones.

Better interaction between academia, the drug industry, and health authorities should develop in order to have better access to bio-collections and to results of trials, specifically negative ones [14]. I do hope you will enjoy reading this series of articles and that they will stimulate new ideas for improvement.

## Note

This article is part of the collection ‘*Why is there persistent disease despite aggressive therapy of rheumatoid arthritis?*’, edited by Pierre Miossec. Other articles in this series can be found at http://arthritis-research.com/series/residual.

## Abbreviations

HLA: Human leukocyte antigen; MTX: Methotrexate; RA: Rheumatoid arthritis; Th17: T helper 17; TNF: Tumor necrosis factor.
